# Rotational abnormalities in dysplastic hips and how to predict acetabular torsion

**DOI:** 10.1007/s00330-022-08895-0

**Published:** 2022-06-09

**Authors:** Carsten Y. W. Heimer, Friedemann Göhler, J. Turner Vosseller, Sebastian Hardt, Carsten Perka, Henrik C. Bäcker

**Affiliations:** 1 Department of Orthopaedic Surgery and Traumatology, Charité Berlin, University Hospital, Chariteplatz 1, 10117 Berlin, Germany; 2 Department of Radiology, Charité Berlin, University Hospital, Chariteplatz 1, 10117 Berlin, Germany; 3 Jacksonville Orthopaedic Institute, San Marco Blvd, Jacksonville, FL 32207 USA

**Keywords:** Radiography CT, Periacetabular, Hip, Dysplasia, Torsion

## Abstract

**Objectives:**

The aim of this study was to investigate the degree to which conventional radiography can represent the acetabular and femoral rotational alignment profile between dysplastic and borderline-dysplastic hips.

**Methods:**

A retrospective trial was conducted including 56 borderline-dysplastic and dysplastic hips at a mean age of 28.9 years (range from 18 to 46). Inclusion criteria consisted of symptomatic patients with hip dysplasia undergoing 2-dimensional radiography as well as computed tomography. On radiography, the lateral center edge angle, acetabular hip index, hip lateralization index, acetabular index angle, and the Sharp angle were measured, and the presence of a crossover sign was noted. In computed tomography, the full rotational profile of the lower limb was measured.

**Results:**

Significant correlations were observed in the overall analysis between the anteversion of the acetabulum and the hip lateralization index (mean 0.56, coefficient of regression (CoR) −32.35, *p* = 0.011) as well as the acetabular index angle with a mean of 11.50 (CoR 0.544, *p* = 0.018). Similar results were found in the subgroup of dysplastic hips with an acetabular index angle of 13.9 (*p* = 0.013, CoR 0.74). For the borderline-dysplastic group, no significant correlations between the pelvis radiography and rotational CT were seen.

**Conclusion:**

Although the femoral and acetabular torsion cannot be predicted from x-rays, the anteversion of the acetabulum correlates with the acetabular index angle, the hip lateralization index, and eventually the beta angle in dysplastic hips. For borderline-dysplastic hips, such results did not show up, which strongly illustrates the need for computed tomography in these cases.

**Key Points:**

*• Much of the current literature focuses on rotational alignment especially with respect to the femur and tibia in healthy patients, although little is known about the acetabular, femoral, and tibial torsion in dysplastic hips.*

*• This is the first study showing significant correlations between the anteversion of the acetabulum and the hip lateralization index as well as the acetabular inclination angle. Also, it is the first study to provide a mechanism for estimation of the torsion of the acetabulum with plain radiography in dysplastic hips.*

*• In borderline-dysplastic hips, no significant correlation was found, which raises the question if a simple x-ray has enough validity to address the acetabular deformity with surgery.*

## Background

Developmental dysplasia of the hip (DDH) is an orthopedic disease with a variation in incidence among different ethnicities [1]. Even though Germany started screening infants in 1996 using ultrasonography at the latest at the age of 5 weeks, Partenheimer et al reported that up to 18% of infants with severe hip dysplasia are under- or misdiagnosed [2]. If conservative treatment fails, surgery might be required in adolescence or adulthood [3] in an effort to both improve function and also decrease the risk for future degenerative change [4]. Indeed, DDH is one of the leading causes of secondary osteoarthritis of the hip [5].

For diagnosis, plain radiographs are performed, including two conventional radiographs—one of the pelvis, in anteroposterior and one axial view of the affected hip in abduction [6, 7]. The relevant radiographic measurements include the lateral center edge angle, anterior center edge angle, acetabular hip index, and acetabular index angle (AIA) [8]. For more precise measurements, a computed tomography (CT) can be requested to assess the femoral and acetabular torsion [9]. However, this is not performed routinely and data on the rotational profile of the CT in correlation to plain radiography is lacking in the literature, especially in DDH or borderline hips [10–14]. Some authors have provided a comparison between the lesser trochanter size on plain radiographs to the femoral version on a CT [15], although further description is largely lacking. Furthermore, there is no objective definition for the diagnosis of DDH based on these rotational values. This lack of a clear cut-off can sometimes cloud decision-making. Given the significance of the surgical intervention required, whether it be the triple osteotomy of Tönnis [16] or the Bernese periacetabular osteotomy (PAO) of Ganz [7] both of which correct the pathological torsion and improve femoral head coverage, more clarity in terms of decision-making would be advantageous.

Therefore, the aim of this study was to assess how torsion of the acetabulum and femur, as assessed by CT, correlated with and can be estimated from conventional measurements on plain radiography in dysplastic hips compared to borderline-dysplastic hips.

## Methods

A retrospective trial was conducted after internal review board (IRB) approval was obtained from the local ethical committee. Between 2017 and 2019, all patients 18 years of age or older presenting to a major hip preservation center and diagnosed with hip dysplasia or borderline hip dysplasia who underwent CT for the assessment of rotational alignment and subsequent surgery were included in our study. Inclusion criteria consisted of full available medical reports as well as preoperative radiography and CT of the lower limb. Exclusion criteria were incomplete records as well as no accessible CT.

**Fig. 1:**
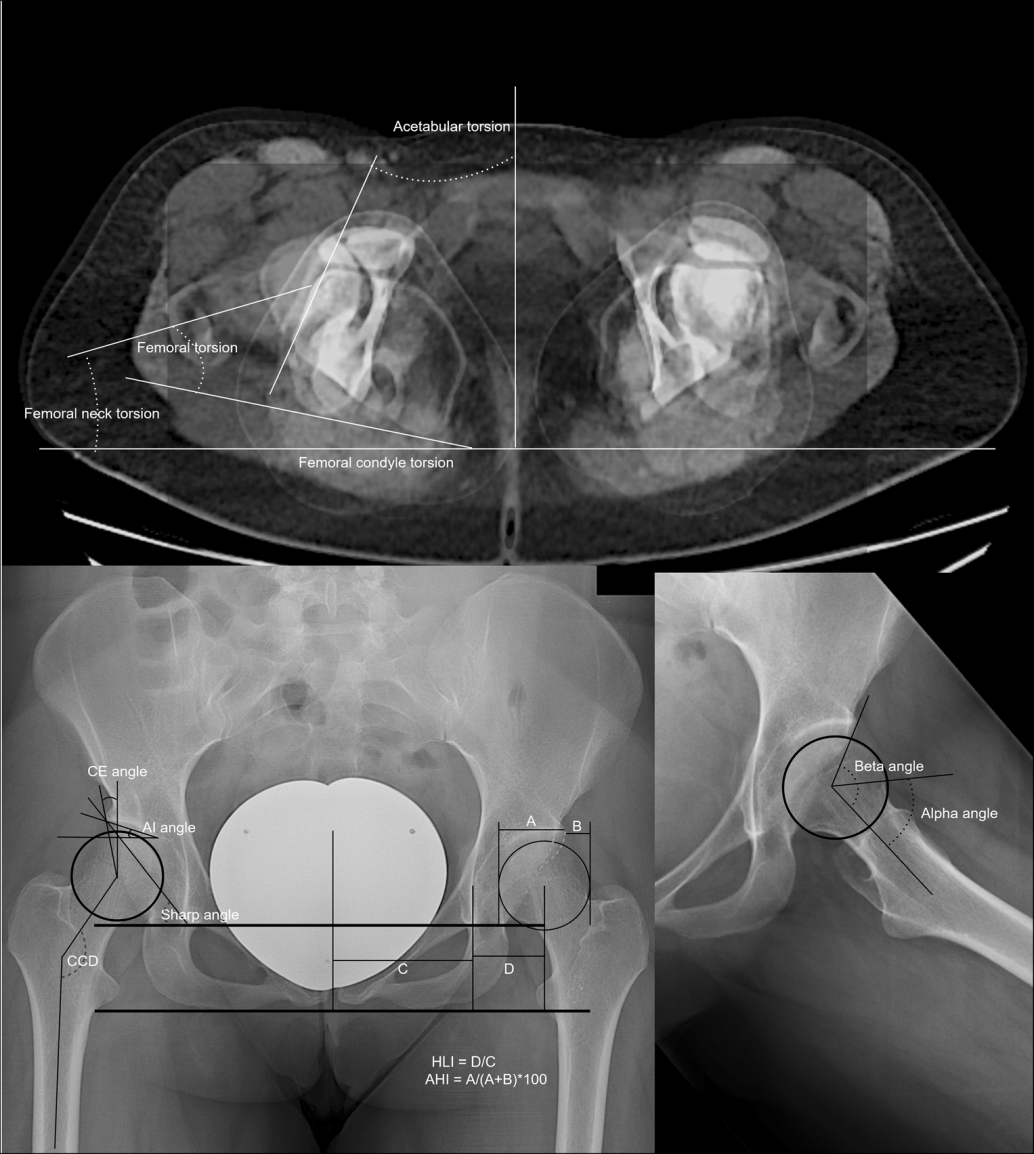
Measurements performed on plain radiograps and in rotational CT

Data on demographics including age, gender, body weight, body height, and body mass index (BMI) were noted. On conventional radiography, common standard measurements were analyzed by a single orthopedic surgery–trained observer with a focus on hip preservation surgery (first author). These included the center-edge (CE) angle, the acetabular index angle, the Sharp angle, the hip lateralization and the acetabular hip index, and the centrum-collum-diaphyseal angle (CCD) on anteroposterior view as well as the alpha and beta angle on axial view. Furthermore, the presence of a crossing-over sign, CAM, or pincer femoroacetabular impingement was identified as illustrated in Fig. 1.

### Image acquisition

All patients underwent a non-enhanced CT of the lower limb, either on a 320-row or on an 80-row CT scanner (Canon Aquilion ONE Vision Edition respectively Canon Aquilion PRIME, Canon Medical Systems). The protocol included a scanogram and a helical acquisition of the lower limb. The scan was performed with 120-KVp tube voltage and automated tube current modulation set low dose mode (standard deviation of 25).

### Image postprocessing

CT images were computed with 0.5 to 1.0mm slice thickness using iterative reconstruction (Adaptive Iterative Dose Reduction (AIDR) 3D standard) and a bone kernel (Filter Convolution (FC) 08-H).

### Measurements in rotational CT

A musculoskeletal fellowship–trained radiologist who was blinded to the diagnosis evaluated the rotational CTs in axial images of the lower limb scan. Acetabular rotation was determined at the level of the acetabular center as an angle between a tangent along the posterior and the anterior acetabular edge and a tangent along the right and left sciatic spina. Angles for measures of femoral torsion, tibial torsion, and tibiofemoral torsion were determined against an image baseline, parallel to the inferior image border.

Femoral torsion was calculated as the difference between femoral neck rotation (angle between a line through femoral neck and femoral head center and image baseline) and the rotation of the femoral condyles (angle between tangent along the posterior condyle border and image baseline).

The tibial torsion was calculated as the difference between tibial plateau rotation (angle between a tangent along the posterior edge of the tibial plateau and image baseline) and the rotation of the upper ankle (angle between a line through the talus and the lateral malleolus and the image baseline).

Furthermore, the tibiofemoral rotation difference was calculated as the difference between the rotation of the femoral condyles and the rotation of the tibial plateau.

Hip dysplasia was defined according to common values, as published in studies for clinical evaluation and orientation and summarized by Tannast et al [17–20]

A borderline-dysplastic hip was defined as a CE angle between 20 and 24.9°, a Sharp angle between 39 and 42°, or the presence of a crossing-over sign with normal values. A dysplastic hip was defined as a CE-angle of less than 20°, the acetabular index angle (AIA) greater than 10°, the Sharp angle greater than 42°, or the acetabular hip index (AHI) greater than 25°. All reference values are summarized in Table 1 [17, 18].

**Table 1:** The values of the definition of the dysplasia, borderline and normal in a table based on Henle et al [17] and added with the Sharp angles as in Mannava et al [16]

Parameter in plain radiography	Dysplasia	Borderline-dysplasia	Normal	FAI
CE-Angle [°]	< 20	20–24.9	25–39	> 39
AIA [°]	> 10		0–10	< 0
AHI [%]	> 25		10–26	< 10
Sharp angle [°]	> 42	39–42	33–38.9	

For normal rotational measurements, the acetabular and the femoral torsions were defined to be between 10 and 25° [21, 22].

### Statistical analysis

For statistical analysis, we used Microsoft® Excel (version 16.36) and IBM SPSS Statistics 26 Core System. Normally distributed continuous variables are presented with the mean and standard deviation of the mean (SD). A mixed model was applied, because it encounters the dependent variable of the person. The level of significance was set to * *p* value ≤ 0.05.

## Results

Between 2017 and 2019, 99 patients presented with borderline or dysplastic hips to our center. In 38 patients a rotational computed tomography was obtained of which 34 patients met inclusion criteria with 56 borderline or dysplastic hips. Females made up the majority of patients (89.3%, or 50/56) and the overall mean age was 28.9 ± 7.8 years (range 18 to 40 years). The average height was 170.2 cm (5’ 6”), at a mean body weight of 68.1 kg (150.1 lbs) and a mean BMI of 23.6 kg/m^2^. Hereby, significant differences (*p* < 0.01) were observed in gender with a mean age of 34.5 ± 4.5 years in males vs. 28.2 ± 7.9 years in females, a body height of 184.8 ± 3.3 cm (6’ 0”) vs. 167.9 ± 6.8 cm (5’ 5”), a body weight of 83.5 ± 9.5 kg (184.1 lbs) vs. 66.2 ± 10.8 kg (145.9 lbs), and a mean BMI of 24.5 ± 3.0 kg/m^2^ vs. 23.6 ± 4.1 kg/m^2^.

Of the 56 hips, 43 were dysplastic and 13 were borderline-dysplastic. All individual demographics are summarized in Table 2.

**Table 2:** Mean ± standard deviation and (minimum – maximum) for the demographics, the x-ray measurements, and rotational profile in all three groups; *p* value of the two-sided t-test ; * positive values stand for external, negative values for internal rotation; ** positive values stand for anteversion, negative values for a retroversion *p* value of the two-sided t-test. Significant correlations were defined as p<0.05 (in bold)

	Overall	Dysplasia	Borderline-dysplasia	*p* value
X-ray measurements				
Gender (female/male)	50/6	40/3	10/3	
Side (right/left)	29/27	22/21	7/6	
Age	28.86 ± 7.84	29.49 ± 8.32	28.00 ± 6.31	
Height [cm]	170.24 ± 8.31	169.88 ± 7.29	171.55 ± 11.64	
Weight [kg]	68.08 ± 11.83	67.18 ± 10.68	71.36 ± 15.49	
BMI	23.55 ± 4.22	23.41 ± 4.36	24.05 ± 3.82	
CE angle [°]	22.48 ± 5.77 (5.2–34.6	**20.92 ± 5.37** **(5.2–28.1)**	**27.64 ± 3.74** **(22.8–34.6)**	**< 0.001**
AIA [°]	11.50 ± 5.22 (1.4–24.7)	**13.09 ± 4.8** **(1.9–24.7)**	**6.26 ± 2.38** **(1.4–9.7)**	**< 0.001**
Sharp angle [°]	42.33 ± 3.66 (35.1–52.3)	**42.99 ± 3.86** **(35.1–52.3)**	**40.18 ± 1.67** **(36.0–42.0)**	**< 0.001**
Hip lat.	0.56 ± 0.07 (0.428–0.717)	0.56 ± 0.06 (0.428–0.676)	0.56 ± 0.07 (0.5–0.717)	0.971
AHI	23.69 ± 7.03 (9.47–45.56)	**25.29 ± 6.87** **(13.64–45.56)**	**18.42 ± 4.72** **(9.47–24.83)**	**< 0.001**
CCD angle [°]	133.53 ± 5.78 (120.47–147.80)	133.76 ± 5.89 (120.47–147.80)	132.75 ± 5.55 (121.0–139.6)	0.627
Alpha angle [°]	57.52 ± 7.64 (40.8–74.2)	57.30 ± 7.78 (40.8–74.2)	58.33 ± 7.53 (46.3–69.8)	0.743
Beta angle [°]	100.68 ± 10.99 (77.8–119.6)	101.18 ± 11.21 (77.8–119.6)	98.88 ± 10.68 (86.2–118)	0.606
Rotational CT measurements				
Torsion of the acetabulum [°] *	18.78 ± 5.62 (6 - 32)	18.50 ± 5.93 (6 - 32)	19.69 ± 4.59 (12 - 29)	0.509
Torsion of the femoral neck [°] *	16.48 ± 11.11 (−28 - 34)	17.07 ± 11.27 (−28 to 34)	14.54 ± 10.78 (−11 to 30)	0.477
Torsion of the femoral condyles [°] *	−13.16 ± 9.51 (−31 to 12)	−13.16 ± 10.37 (−31 to 12)	−13.15 ± 6.16 (−24–[−2])	0.998
Femoral torsion [°] **	28.84 ± 12.82 (3–57)	29.65 ± 13.44 (3–57)	26.15 ± 10.51 (12–43)	0.394
Torsion of the tibial plateau [°] *	−7.57 ± 10.14 (−25 to 18)	−7.12 ± 10.96 (−25 to 18)	−9.08 ± 6.86 (−25 to 0)	0.546
Femoral-tibial torsion difference[°]	6.16 ± 5 (0–30)	**6.74 ± 5.34** **(0–30)**	**4.23 ± 3.09** **(0–10)**	**0.040**
Torsion of the upper ankle [°] *	29.24 ± 10.53 (7–53)	29.53 ± 10.83 (7–53)	28.27 ± 9.82 (12–43)	0.732
Tibial torsion [°] **	37.24 ± 7.74 (19–60)	37.18 ± 7.4 (19–60)	37.45 ± 9.21 (19–53)	0.92
Torsion of the whole leg (torsion of the femoral neck - torsion of the upper ankle)	−13.22 ± 13.87 (−39 to 21)	−13.16 ± 14.58 (−39 to 21)	−13.45 ± 11.69 (−30 to 11)	0.951

For standard DDH measurements on plain radiography, the acetabular index angle and CE angle were more abnormal for the hip dysplasia group compared to the borderline-dysplasia group at 13.1° ± 4.80 compared to 6.3° ± 2.38 and 20.9° ± 5.37 vs. 27.6° ± 3.74 (all *p* < 0.001), respectively. Additionally, the acetabular hip index and sharp angle were significantly different. All individual findings are summarized in Table 2. For the rotational measurements, only significant differences in the femoral-tibial rotational difference were found with 6.7° ± 5.34 vs. 4.2° ± 3.09 (*p* = 0.04). Furthermore, Table 2 shows all findings measured on computed tomography.

### Overall

After applying the mixed model, significant correlations between the CE-angle and the acetabular index angle, the Sharp angle as well as the acetabular hip index were found. For the torsion of the acetabulum (18.78° ± 5.62°), a significant correlation was observed with regard to the acetabular index angle at a mean of 11.50° ± 5.22 and a coefficient of regression (CoR) of 0.544 (*p* = 0.018, CI [0.096, 0.992]) and the hip lateralization index (HLI) at a mean of 0.56 ± 0.07 (regression coefficient of −32.35 (*p* = 0.011, CI [−56.836, −7.864])). Therefore, a decrease of 0.1 in hip lateralization index leads to an increase in anteversion of the acetabulum by 3.24° (Table 3). Other significant findings were identified between the femoral neck torsion (16.48° ± 11.11°) and the beta angle, at a mean of 100.68° ± 10.99°, and additionally, between the torsion of the acetabulum and the beta angle, at a mean of 57.52° ± 7.64°. All findings are illustrated in Table 3.

**Table 3:** Correlations between the different computed tomography findings, CE angle, and the radiographic measurements among all hips. All values are presented with the regression coefficient, *p* value, and confidence interval. Significant correlations were defined as p<0.05 (in bold)

Overall	CE angle	Acetabular index angle	Hip lateralization index	Sharp angle	Acetabular hip index	Alpha angle	Beta angle	CCD-angle
Torsion of the acetabulum	0.479	**0.544**	**–32.35**	–0.022	0.053	**–0.298**	–0.072	–0.113
	*p* = 0.140	***p* = 0.018**	***p* = 0.011**	*p* = 0.941	*p* = 0.833	***p* = 0.017**	*p* = 0.446	*p* = 0.354
	CI [–0.165; 1.123]	**CI [0.096; 0.992]**	**CI [–56.836; –7.864]**	CI [–0.601; 0.558]	CI [–0.453; 0.56]	**CI [–0.538; –0.058]**	CI [–0.262; 0.118]	CI [–0.355; 0.129]
Femoral torsion	–1.072	–0.254	–14.296	–0.682	–0.221	–0.089	–0.318	0.524
	*p* = 0.170	*p* = 0.632	*p* = 0.634	*p* = 0.342	*p* = 0.719	*p* = 0.752	*p* = 0.124	*p* = 0.083
	CI [–2.626; 0.483]	CI [–1.313; 0.805]	CI [–74.262; 45.670]	CI [–2.111; 0.746]	CI [–1.452; 1.01]	CI [–0.657; 0.479]	CI [–0.092; 0.729]	CI [–0.072; 1.119]
Femoral neck torsion	–0.855	0.172	38.326	–0.096	–0.64	–0.239	**0.351**	0.369
	*p* = 0.174	*p* = 0.700	*p* = 0.136	*p* = 0.873	*p* = 0.213	*p* = 0.254	***p* = 0.025**	*p* = 0.141
	CI [–2.111; 0.401]	CI [–0.721; 1.065]	CI [–12.519; 89.171]	CI [–1.290; 1.099]	CI [–1.66; 0.380]	CI [–0.656; 0.179]	**CI [0.046; 0.655]**	CI [–0.127; 0.865]
Torsion of the femoral condyles	–0.289	0.199	23.282	–0.023	–0.47	–0.104	0.059	0.086
	*p* = 0.501	*p* = 0.558	*p* = 0.236	*p* = 0.960	*p* = 0.211	*p* = 0.644	*p* = 0.717	*p* = 0.635
	CI [–1.158; 0.581]	CI [–0.481; 0.879]	CI [–15.781; 62.346]	CI [–0.92; 0.874]	CI [–1.218; 0.278]	CI [–0.555; 0.348]	CI [–0.272; 0.391]	CI [–0.279; 0.452]
Tibial torsion	–1.074	0.146	–3.416	–0.289	–0.73	–0.270	0.126	0.362
	*p* = 0.086	*p* = 0.687	*p* = 0.874	*p* = 0.581	*p* = 0.109	*p* = 0.172	*p* = 0.335	*p* = 0.100
	CI [–2.307; 0.158]	CI [–0.581; 0.873]	CI [–46.922; 40.091]	CI [–1.337; 0.759]	CI [–1.631; 0.171]	CI [–0.665; 0.124]	CI [–0.137; 0.389]	CI [–0.073; 0.797]
Torsion of the tibial plateau	–0.731	0.050	13.266	0.089	–0.703	0.019	–0.095	0.066
	*p* = 0.228	*p* = 0.904	*p* = 0.577	*p* = 0.875	*p* = 0.150	*p* = 0.938	*p* = 0.581	*p* = 0.780
	CI [–1.940; 0.478]	CI [–0.786; 0.887]	CI [–34.193; 60.725]	CI [–1.037; 1.215]	CI [–1.670; 0.264]	CI [–0.467; 0.505]	CI [–0.442; 0.253]	CI [–0.403; 0.534]
Torsion of the upper ankle	–0.587	0.165	–18.707	–0.139	–0.375	–0.427	0.004	0.330
	*p* = 0.299	*p* = 0.685	*p* = 0.460	*p* = 0.804	*p* = 0.422	*p* = 0.084	*p* = 0.983	*p* = 0.148
	CI [–1.730; 0.556]	CI [–0.649; 0.978]	CI [–69.397; 31.983]	CI [–1.258; 0.981]	CI [–1.312; 0.562]	CI [–0.916; 0.062]	CI [–0.353; 0.346]	CI [–0.123; 0.783]
Femoral-tibial torsion difference	–0.12	–0.149	–14.401	–0.080	0.031	0.154	–0.137	0.029
	*p* = 0.769	*p* = 0.519	*p* = 0.256	*p* = 0.806	*p* = 0.915	*p* = 0.225	*p* = 0.124	*p* = 0.826
	CI [–0.936; 0.696]	CI [–0.614; 0.315]	CI [–39.850; 11.048]	CI [–0.734; 0.573]	CI [–0.547; 0.608]	CI [–0.1; 0.409]	CI [–0.314; 0.039]	CI [–0.240; 0.299]
CE angle		**–0.195**	1.460	**–0.465**	**–0.51**	0.034	**–0.32**	0.032
		***p* = 0.009**	*p* = 0.718	***p*<0.001**	***p*<0.001**	*p* = 0.793	***p* = 0.002**	*p* = 0.462
		**CI [–0.338; –0.051]**	CI [–6.704; 9.624]	**CI [–0.642; –0.288]**	**CI [–0.639; –0.380]**	CI [–0.230; 0.299]	**CI [–0.507; –0.132]**	CI [–0.056; 0.120]

### Dysplastic hips

For dysplastic hips, only a few significant correlations were noted. The highest correlations were observed between the torsion of the acetabulum and the acetabular index angle, with a CoR of 0.74 (CI [0.217, 1.262]) and *p* = 0.007, and the hip lateralization index with a regression coefficient of −35.137 (CI [−62.418, −7.856] and a *p* = 0.013. Other significant findings included statistical correlations for tibial plateau torsion and the acetabular hip index with a CoR of −1.261 (*p* value 0.049, CI [−2.516, −0.007]). In addition, the femoral neck torsion correlated with the CCD angle and the femoral-tibial rotation difference with the beta angle, as indicated in Table 4.

**Table 4:** Correlations between the different computed tomography findings, CE angle, and the radiographic measurements among all dysplastic hips. All values are presented with the regression coefficient, *p* value, and confidence interval. Significant correlations were defined as p<0.05 (in bold)

Dysplastic hips	CE angle	Acetabular index angle	Hip lateralization index	Sharp angle	Acetabular hip index	Alpha angle	Beta angle	CCD-angle
Torsion of the acetabulum	0.598	**0.74**	**–35.137**	0.054	0.021	–0.265	–0.082	–0.073
	*p* = 0.122	***p* = 0.007**	***p* = 0.013**	*p* = 0.861	*p* = 0.943	*p* = 0.070	*p* = 0.470	*p* = 0.585
	CI [–0.173, 1.369]	**CI [0.217, 1.262]**	**CI [–62.418, –7.856]**	CI [–0.565, 0.673]	CI [–0.579, 0.622]	CI [–0.554, 0.023]	CI [–0.312, 0.148]	CI [–0.343, 0.197]
Femoral torsion	0.005	–0.634	–10.281	–0.685	0.728	0.179	0.289	0.604
	*p* = 0.996	*p* = 0.319	*p* = 0.767	*p* = 0.394	*p* = 0.341	*p* = 0.574	*p* = 0.214	*p* = 0.089
	CI [–1.953, 1.964]	CI [–1.906, 0.638]	CI [–80.1, 59.537]	CI [–2.296, 0.926]	CI [–0.802, 2.258]	CI [–0.47, 0.828]	CI [–0.179, 0.757]	CI [–0.096, 1.304]
Femoral neck torsion	–0.683	0.332	23.935	–0.328	–0.588	–0.053	0.286	**0.597**
	*p* = 0.391	*p* = 0.524	*p* = 0.403	*p* = 0.619	*p* = 0.350	*p* = 0.812	*p* = 0.086	***p* = 0.043**
	CI [–2.299, 0.934]	CI [–0.714, 1.378]	CI [–33.479, 81.349]	CI [–1.652, 0.997]	CI [–1.846, 0.671]	CI [–0.508, 0.402]	CI [–0.044, 0.617]	**CI [0.020, 1.173]**
Torsion of the femoral condyles	–0.656	0.487	11.538	–0.127	–0.878	–0.163	0.043	0.151
	*p* = 0.235	*p* = 0.267	*p* = 0.623	*p* = 0.812	*p* = 0.085	*p* = 0.549	*p* = 0.829	*p* = 0.489
	CI [–1.781, 0.47]	CI [–0.389, 1.362]	CI [–35.798, 58.873]	CI [–1.206, 0.952]	CI [–1.884, 0.127]	CI [–0.715, 0.389]	CI [–0.363, 0.45]	CI [–0.292, 0.593]
Tibial torsion	–0.705	0.189	11.074	–0.005	–0.489	–0.307	0.092	0.046
	*p* = 0.406	*p* = 0.654	*p* = 0.654	*p* = 0.993	*p* = 0.375	*p* = 0.184	*p* = 0.555	*p* = 0.859
	CI [–2.431, 1.021]	CI [–0.672, 1.051]	CI [–39.941, 62.089]	CI [–1.226, 1.215]	CI [–1.596, 0.619]	CI [–0.771, 0.157]	CI [–0.226, 0.411]	CI [–0.482, 0.575]
Torsion of the tibial plateau	–1.185	0.396	–1.032	0.086	**–1.261**	0.149	–0.197	0.151
	*p* = 0.128	*p* = 0.452	*p* = 0.971	*p* = 0.896	***p* = 0.049**	*p* = 0.603	*p* = 0.339	*p* = 0.594
	CI [–2.741, 0.370]	CI [–0.66, 1.452]	CI [–58.814, 56.751]	CI [–1.24, 1.412]	**CI [–2.516, –0.007]**	CI [–0.434, 0.733]	CI [–0.613, 0.220]	CI [–0.42, 0.721]
Torsion of the upper ankle	–0.685	0.297	–17.376	–0.09	–0.452	–0.39	–0.147	0.247
	*p* = 0.347	*p* = 0.553	*p* = 0.561	*p* = 0.891	*p* = 0.452	*p* = 0.156	*p* = 0.460	*p* = 0.365
	CI [–2.178, 0.808]	CI [–0.713, 1.307]	CI [–77.674, 42.923]	CI [–1.41, 1.231]	CI [–1.663, 0.759]	CI [–0.939, 0.159]	CI [–0.551, 0.258]	CI [–0.305, 0.798]
Femoral-tibial torsion difference	0.055	–0.358	–17.671	–0.14	0.197	**0.312**	–0.194	0.062
	*p* = 0.921	*p* = 0.200	*p* = 0.236	*p* = 0.710	*p* = 0.584	***p* = 0.034**	*p* = 0.055	*p* = 0.691
	CI [–1.059, 1.169]	CI [–0.918, 0.202]	CI [–47.859, 12.516]	CI [–0.9, 0.620]	CI [–0.53, 0.924]	**CI [0.026, 0.599]**	CI [–0.393, 0.005]	CI [–0.260, 0.384]
CE angle		–0.146	–1.406	**–0.446**	**–0.514**	–0.032	**–0.339**	0.024
		*p* = 0.065	*p* = 0.742	***p*<0.001**	***p*<0.001**	*p* = 0.792	***p* = 0.001**	*p* = 0.592
		CI [–0.301, 0.01]	CI [–10.000, 7.188]	**CI [–0.614, –0.277]**	**CI [–0.642, –0.385]**	CI [–0.280, 0.216]	**CI [–0.516, –0.163]**	CI [–0.067, 0.115]

### Borderline-dysplastic hips

When looking for borderline-dysplastic hips, only three significant correlations have been found. These included, like in the dysplastic group, the CE-angle with the acetabular hip index (CoR −0.69, *p* = 0.022, CI [−1.243; −0.136]) and the femoral-tibial rotation difference with the beta angle but with a reversed coefficient of regression (−0.434, *p* = 0.009, CI [−0.701; −0.167]). All results are summarized in Table 5.

**Table 5:** Correlations between the different computed tomography findings, CE angle and the radiographic measurements among all borderline-dysplastic hips. All values are presented with the regression coefficient, *p* value, and confidence interval. Significant correlations were defined as p<0.05 (in bold)

Borderline hips	CE angle	Acetabular index angle	Hip lateralization index	Sharp angle	Acetabular hip index	Alpha angle	Beta angle	CCD-angle
Torsion of the acetabulum	−0.593	−0.879	−39.539	−0.985	0.084	−0.523	−0.005	0.008
	*p* = 0.433	*p* = 0.220	*p* = 0.207	*p* = 0.349	*p* = 0.903	*p* = 0.068	*p* = 0.977	*p* = 0.979
	CI [−2.320; 1.134]	CI [−2.449; 0.690]	CI [−107.917; 28.84]	CI [−3.359; 1.388]	CI [−1.518; 1.685]	CI [−1.102; 0.057]	CI [−0.414; 0.404]	CI [−0.719; 0.735]
Femoral torsion	−4.477	0.2	16.845	−2.197	−3.088	−1.061	0.332	0.908
	*p* = 0.051	*p* = 0.899	*p* = 0.798	*p* = 0.357	*p* = 0.078	*p* = 0.123	*p* = 0.449	*p* = 0.337
	CI [−8.981; 0.026]	CI [−3.507; 3.907]	CI [−136.993; 170.683]	CI [−7.589; 3.194]	CI [−6.653; 0.477]	CI [−2.531; 0.409]	CI [−0.706; 1.368]	CI [−1.219; 3.034]
Femoral neck torsion	−3.297	−2.432	113.94	0.014	−2.134	−0.956	0.48	0.351
	*p* = 0.115	*p* = 0.149	*p* = 0.111	*p* = 0.995	*p* = 0.182	*p* = 0.135	*p* = 0.261	*p* = 0.692
	CI [−7.670; 1.075]	CI [−6.031; 1.168]	CI [−35.435; 263.314]	CI [−5.222; 5.251]	CI [−5.596; 1.327]	CI [−2.334; 0.422]	CI [−0.492; 1.452]	CI [−1.717; 2.419]
Torsion of the femoral condyles	−0.104	−0.452	57.659	1.034	−0.246	0.105	0.149	−0.197
	*p* = 0.911	*p* = 0.591	*p* = 0.176	*p* = 0.431	*p* = 0.770	*p* = 0.815	*p* = 0.643	*p* = 0.630
	CI [−2.290; 2.081]	CI [−2.400; 1.497]	CI [−35.750; 151.069]	CI [−1.976; 4.045]	CI [−2.221; 1.729]	CI [−0.992; 1.203]	CI [−0.626; 0.923]	CI [−1.169; 0.776]
Tibial torsion	−1.739	0.226	−38.185	−1.713	−0.641	−0.121	0.244	1.136
	*p* = 0.239	*p* = 0.824	*p* = 0.506	*p* = 0.314	*p* = 0.603	*p* = 0.852	*p* = 0.518	*p* = 0.075
	CI [−5.287; 1.809]	CI [−2.429; 2.881]	CI [−184.477; 108.107]	CI [−5.913; 2.488]	CI [−3.796; 2.514]	CI [−2.018; 1.776]	CI [−0.817; 1.305]	CI [−0.197; 2.469]
Torsion of the tibial plateau	0.173	−1.049	33.884	0.05	0.134	−0.374	0.208	−0.348
	*p* = 0.876	*p* = 0.275	*p* = 0.386	*p* = 0.970	*p* = 0.878	*p* = 0.449	*p* = 0.546	*p* = 0.514
	CI [−2.423; 2.768]	CI [−3.185; 1.088]	CI [−54.776; 122.544]	CI [−3.058; 3.157]	CI [−1.920; 2.189]	CI [−1.545; 0.797]	CI [−0.618; 1.034]	CI [−1.573; 0.878]
Torsion of the upper ankle	−3.277	−1.420	77.762	−1.661	−2.339	−1.414	**0.912**	1.070
	*p* = 0.242	*p* = 0.473	*p* = 0.474	*p* = 0.585	*p* = 0.335	*p* = 0.066	***p* = 0.047**	*p* = 0.291
	CI [−10.122; 3.568]	CI [−6.431; 3.591]	CI [−200.668; 356.193]	CI [−9.744; 6.422]	CI [−8.277; 3.599]	CI [−3.001; 0.174]	**CI [0.024; 1.800]**	CI [−1.641; 3.782]
Femoral-tibial torsion difference	−0.007	−0.362	−11.453	−0.618	−0.009	**−0.434**	0.03	−0.066
	*p* = 0.990	*p* = 0.521	*p* = 0.657	*p* = 0.470	*p* = 0.987	***p* = 0.009**	*p* = 0.701	*p* = 0.799
	CI [−1.483; 1.468]	CI [−1.687; 0.963]	CI [−88.767;65.861]	CI [−2.603; 1.367]	CI [−1.336; 1.318]	**CI [−0.701; −0.167]**	CI [−0.158; 0.218]	CI [−0.724; 0.591]
CE angle		−0.344	18.674	−0.324	**−0.69**	0.051	−0.073	0.179
		*p* = 0.315	*p* = 0.200	*p* = 0.542	***p* = 0.022**	*p* = 0.826	*p* = 0.658	*p* = 0.254
		CI [−1.096; 0.408]	CI [−12.523; 49.871]	CI [−1.518; 0.87]	**CI [−1.243; −0.136]**	CI [−0.512; 0.613]	CI [−0.469; 0.324]	CI [−0.162; 0.519]

Based on our findings on acetabular torsion and correlation to the AIA and hip lateralization angle, we developed a formula to estimate rotation from plain radiography. The AIA, the hip lateralization index, the beta angle, and the CE angle were used in the following formula.

$$ \mathrm{Torsion}\ \mathrm{of}\ \mathrm{the}\ \mathrm{acetabulum}\ \left(\pm {4}^{{}^{\circ}}\right)=34.72+0.479\times \mathrm{CE}+0.544\times \mathrm{AIA}+\left(-32.35\right)\times \mathrm{HLI}+\left(-0.298\right)\times \mathrm{alpha}-\mathrm{angle} $$

Hereby, the constant term results from the mixed model of the torsion of the acetabulum in relation to the plain radiography for the overall cohort. Similarly, the indices result from the individual mixed model.

Application of this formula in the current study matched perfectly with the rotational profile in 70% of cases. In a further 6 patients, the acetabular torsion varied ± 8° (89%) whereas in the remaining cases, an excessive anteversion of 28° and higher was found. This formula was not found to apply to borderline-dysplastic hips.

**Fig. 2:**
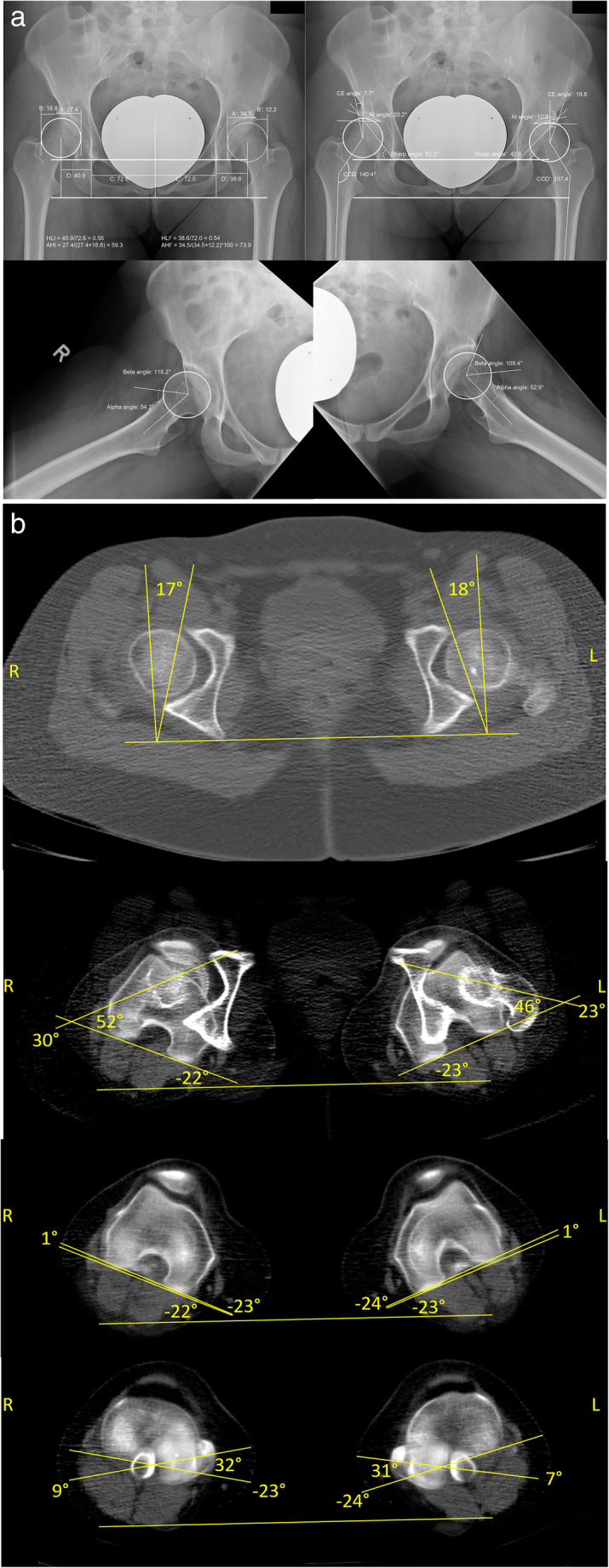
Radiographic Findings in a *dysplastic hip patient* **a** one of the pelvis, in anteroposterior and axial views **b** rotational alignment of the lower extremity; when applying our formula, the estimated acetabular torsion based on the plain radiographies is the following: Right hip: 34.72 + 0.479*7.7 (CE-angle) + 0.544*20.2 (AIA) + (−32.35)*0.56 (HLI) + (−0.298)*54.2 (alpha-angle) = *15.1°*. Left hip: 34.72 + 0.479*18.8 (CE-angle) + 0.544*12.3 (AIA) + (−32.35)*0.54 (HLI) + (−0.298)*52.9 (alpha-angle) = ***17.2°***

**Fig. 3:**
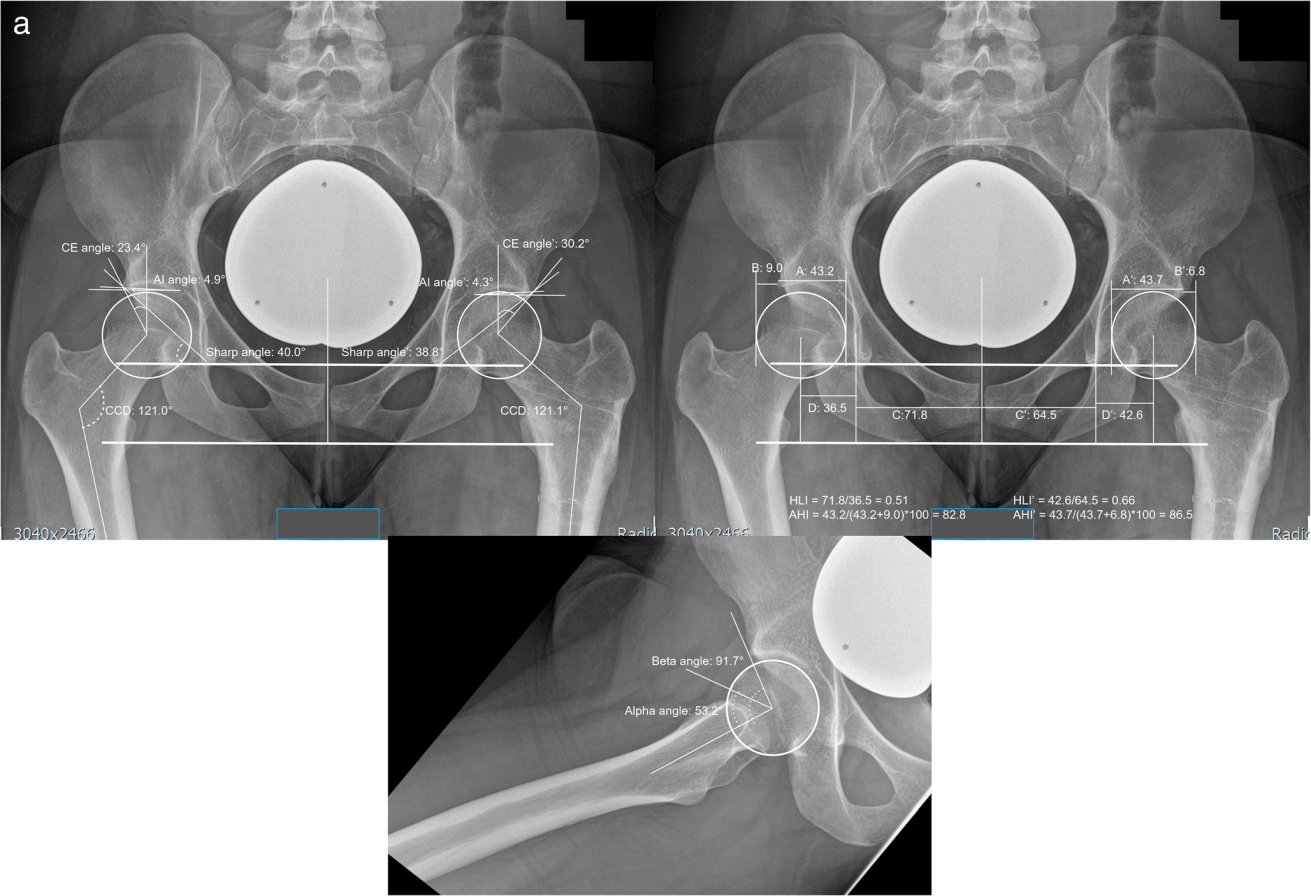

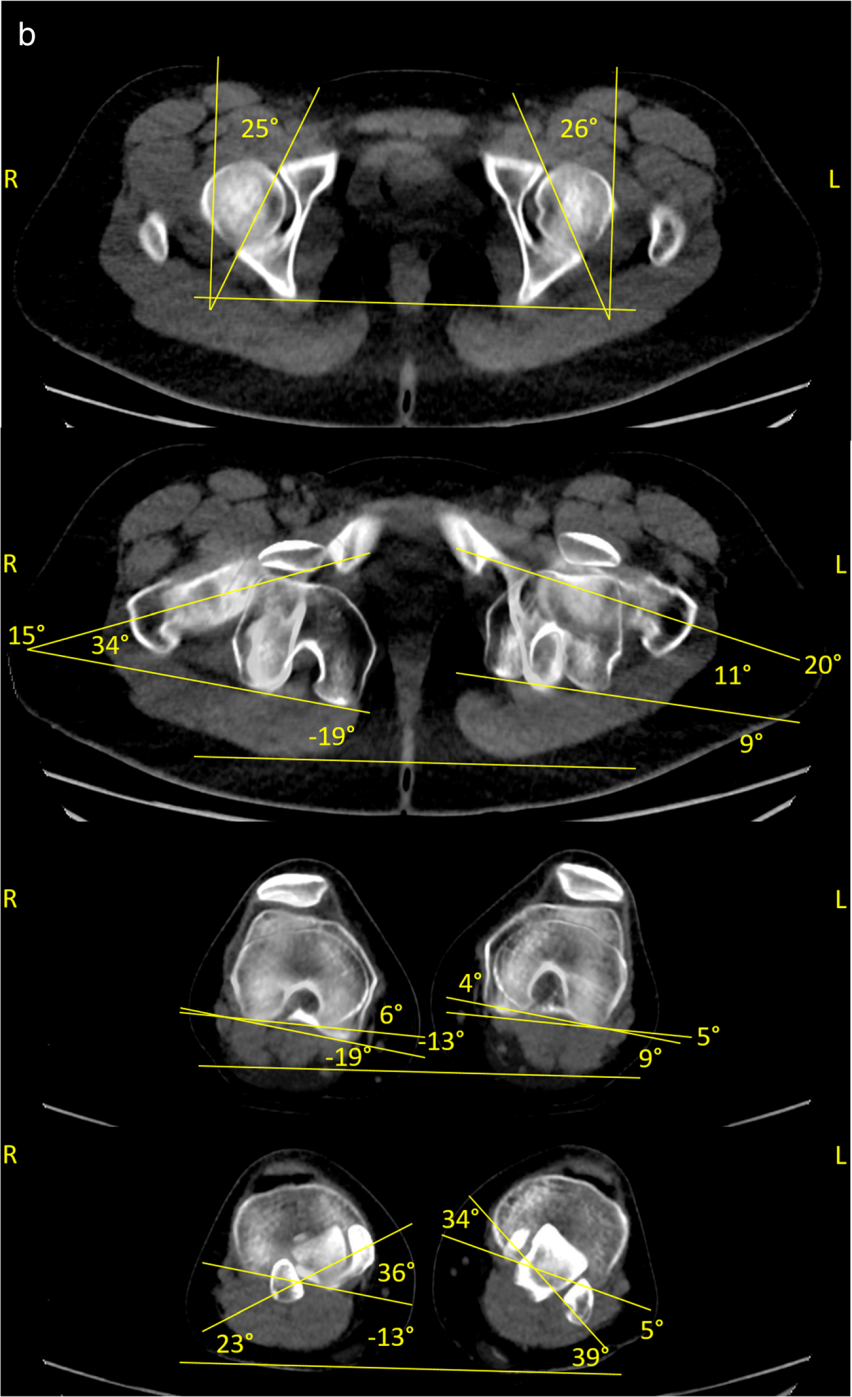
Radiographic findings in a borderline dysplastic hip patient **a** one of the pelvis, in anteroposterior and one axial view **b** rotational alignment of the lower extremity

Measurements of two patients with dysplastic and borderline-dysplastic hips are illustrated in Figs. 2 and 3.

## Discussion

Although the developmental dysplasia of the hip is one of the leading causes of secondary osteoarthritis of the hip, correlation between the rotational alignment of the lower limb (acetabular, femoral, and tibial torsion) and plain radiography of the pelvis is not well understood or described in the literature.

Until today, for rotational measurements of the lower limb, CT is the best diagnostic instrument according to Wissing et al [9]. It allows detailed examination of acetabular, femoral, and tibial torsion. Physical examination showed little correlation between external tibial torsion (57%) and hip rotation (14%) in comparison with the standard of CT [23].

In the diagnosis of hip dysplasia, an AP of the pelvis and an axial view of the affected hip are performed routinely and, in many cases, solely [6; 7]. For the center edge angle, the AIA, Sharp angle, and the beta angle measurements have shown significant correlations on plain radiography; only the alpha angle showed a poor correlation in one rater between the two diagnostic instruments. Therefore, the AIA, Sharp angle, and beta angle are as reliable and important as the center edge angle for the diagnosis of hip dysplasia. However, the correlation to rotational alignment remains unclear [14].

In the literature, rotational measurements of the lower extremity—femoral and tibial torsions—are described primarily in healthy patients [23–26]. Hereby, good validity has been reported between CT and EOS scans [27].

In children, the femoral torsion measurements on 3D models based on biplanar radiographs (BPR) were comparable to MR images, although tibial torsion was not reliable [24].

These studies have not routinely assessed the acetabular version (normal range from 17° ± 6°) [28] or the correlation to plain radiography of the pelvis, especially in dysplastic hips. However, if a periacetabular osteotomy is indicated, not only the femoral head coverage but also the torsional abnormality need to be addressed and therefore be understood in detail [28].

In borderline-dysplastic hips, treatment is less uniform; it is possible that a better understanding of the rotational variation in these deformities may clarify the optimal treatment [29, 30]. A huge variation for the femoral antetorsion can be found in the literature ranging from 10 to 15° [25] with a mean of 12.7° ± 10° [31], including a [32] significant difference between the left and right femur (approx. 4° larger) and a gender difference with 17.8 and 22.7° for female and 15.3 and 21.4° for male healthy femurs [33] As patients age, femoral antetorsion decreases from a toddler to skeletal maturity from 31.1° ± 8.9 at age of 1 to 15.4° ± 7.6 at the age of 16 years. In our cohort, the mean age was 28.9 ± 7.8 years, and so the influence of age was likely not a factor [34].

In femoroacetabular impingement, a high prevalence of combined femoral and tibial torsional abnormalities has been already reported with a mean femoral torsion of 23° and tibial torsion of 29° [32, 35]. Furthermore, decreased femoral torsion showed less flexion and internal rotation in 90° flexion [36].

Even higher values for femoral torsions were observed in our study at 29.65° ± 13.44 compared to the reference values in healthy and FAI patients as reported in the literature [31, 32, 34, 35]. For tibial torsion, the mean was 37.18° ± 7.4 in our cohort. As with femoral torsion, this value was significantly higher compared with normal tibial torsion at 21.6° ± 7.6 (range 4.8 to 39.5) [37, 38]. In addition, a correlation between the femoral neck torsion and the beta angle was observed in all hips. This relates to its configuration, where a greater anteversion results in a higher CCD angle on radiography, causing an increasing beta angle. In contrast, the alpha angle, which expresses the angle of the anterior head-neck congruency between the head center and the acetabular rim, correlated significantly to the acetabular torsion. This correlation may result from an increased anteversion of the acetabulum in borderline- and dysplastic hips [39] and subsequent greater cartilage coverage.

In dysplastic hips, significant differences were found especially for the torsion of the acetabulum in correlation to the AIA and the hip lateralization index which likely results from increased anteversion. As a result, a shorter distance of the lateral edge of the acetabulum to the inferior point of the weight-bearing area of the acetabular sourcil can be observed, which then leads to a steeper angle on plain x-ray. Likewise, the higher the acetabular torsion, the smaller the hip lateralization index gets which originates from a decreased distance between the center of the femoral head to the pelvic teardrop due to both increased anteversion and a relatively ovoid (as opposed to round) femoral head [40]. If the AIA from plain radiography is used to predict the acetabular anteversion, the x-ray must be taken very precisely. The AIA is easy to misinterpret when the hip is rotated or tilted [41]. Based on these findings, we were able to develop a formula to estimate rotation from plain radiography with high accuracy in DDH of 70%. Other findings, included the alpha-angle which was only significant in the overall cohort, not on subgroup analysis (*p* = 0.070 in the dysplastic group, *p* = 0.068 in the borderline-dysplastic group).

When comparing the results of the dysplasia and the borderline dysplasia group, the femoral-tibial rotation difference showed significant correlations to the alpha angle. In dysplastic hips, a positive coefficient of regression was observed (0.312, *p* = 0.034), whereas for borderline-dysplastic hips, this was found to be negative (−0.434, *p* = 0.009).

Since periacetabular osteotomy remains an effective technique to treat symptomatic hip dysplasia with survival of up to 29% of hips 30 years postoperatively [42], a thorough examination is warranted to include the common standard measurements, as well as rotational alignment. An understanding of the correlations between the individual measurements to allow a 3-dimensional reorientation of the acetabulum can allow for more thorough deformity correction. For borderline-dysplastic hips, no significant correlations were found. When considering our results and the existing literature on the unclear indication for PAO or hip arthroscopy [29, 30], further diagnostics are essential including a computed tomography to assess the rotational alignment.

## Limitations

There are several limitations to this study. This retrospective study included 56 hips describing correlations between radiographic measurements and CT findings in dysplastic and borderline-dysplastic hips. Two different observers performed the measurements; the plain radiographs were analyzed by an orthopedic surgery–trained observer with a focus on hip preservation surgery (first author), whereas the computed tomography analysis was performed by a fellowship-trained musculoskeletal radiologist. No consensus reading was performed why we are unable to present the inter-/intraobserver reliability. To further validate the developed formula to estimate the torsion of the acetabulum based on the plain radiography, a lager cohort study is required.

## Conclusion

To our knowledge, this study is the first to analyze the correlations between all torsions of the lower limb with rotational CT and plain radiography in patients with dysplastic and borderline-dysplastic hips. Significant correlations were found especially for the AIA and the hip lateralization index in relation to the rotation of the acetabulum. Based on our findings and the developed formula, this may help to estimate acetabular torsion. As the borderline-dysplastic hips had mostly normal standard measurements on plain radiography with no major differences in rotational CT, further diagnostics to assess the rotational alignment should be performed.

## Abbreviations

AHI: Acetabular hip index
AIA: Acetabular index angle
AIDR: Adaptive iterative dose reduction
CCD: Centrum-collum-diaphyseal
CE: Center-edge
CoR: Coefficient of regression
CT: Computed tomography
DDH: Developmental dysplasia of the hip
HLI: Hip lateralization index
IRB: Internal review board
PAO: Periacetabular osteotomy
SD: Standard deviation of the mean

## Declarations

### Guarantor

The scientific guarantor is Henrik C. Bäcker.

### Statistics and biometry

One of the authors has significant statistical expertise.

### Methodology

• retrospective

• case-control study

• performed at one institution
