# Correction to: Rotational abnormalities in dysplastic hips and how to predict acetabular torsion

**DOI:** 10.1007/s00330-022-09019-4

**Published:** 2022-07-29

**Authors:** Carsten Y. W. Heimer, Friedemann Göhler, J. Turner Vosseller, Sebastian Hardt, Carsten Perka, Henrik C. Bäcker

**Affiliations:** 1 Department of Orthopaedic Surgery and Traumatology, Charité Berlin, University Hospital, Chariteplatz 1, 10117 Berlin, Germany; 2 Department of Radiology, Charité Berlin, University Hospital, Chariteplatz 1, 10117 Berlin, Germany; 3 Jacksonville Orthopaedic Institute, San Marco Blvd, Jacksonville, FL 32207 USA

**Correction to: European Radiology**

10.1007/s00330-022-08895-0

The original version of this article, published on 09 June 2022, unfortunately contained a mistake. The sentence "Two different observers performed the measurements; the plain radiographs were analyzed by an orthopedic-trained surgeon with a focus on hip preservation surgery (first author), whereas the computed tomography analysis was performed by a fellowship-trained musculoskeletal radiologist" was corrected to read "Two different observers performed the measurements; the plain radiographs were analyzed by an orthopedic surgery–trained observer with a focus on hip preservation surgery (first author), whereas the computed tomography analysis was performed by a fellowship-trained musculoskeletal radiologist". The original article has been corrected.

